# Disclosure of congenital cleft lip and palate to Japanese patients: reported patient experiences and relationship to self-esteem

**DOI:** 10.1186/1756-0500-7-924

**Published:** 2014-12-16

**Authors:** Tomoko Omiya, Mikiko Ito, Yoshihiko Yamazaki

**Affiliations:** Community Nursing, Faculty of Nursing, Toho University, 4-16-20 Omori-Nishi, Ota-ku, Tokyo, 143-0015 Japan; Division of Health Promotion Science, Nursing Science, Course of Health Science, Graduate School of Medicine, Osaka University, 1-7 Yamada-oka, Suita, Osaka, 565-0871 Japan; Department of Social Welfare, Faculty of Social Welfare, Nihon Fukushi University, Okuda, Mihama-cho, Chita-gun, Aichi, 470-3295 Japan

**Keywords:** Cleft lip and cleft palate, Self-esteem, Self-stigma, Disclosure, Japan

## Abstract

**Background:**

The present study investigated when and how Japanese people with cleft lip and palate (CL/P) learn that their condition is congenital; the perceived effects of withholding the CL/P diagnosis on patients; and whether the resulting social experience and self-esteem are related. A questionnaire survey was conducted in 71 adults with CL/P recruited through a hospital, a patients’ association, and by snowball sampling.

**Results:**

The participants became aware of their physical difference in childhood, but many reported difficulty in understanding their condition. Participants reported that their families avoided the topic of diagnosis. Participants who understood their condition during childhood rather than in adulthood were significantly more likely to consider this scenario as positive (p < 0.001). Although stigmatising experiences were extremely painful, most patients hid their suffering, making it more difficult to obtain social support. Participants with high self-esteem were more likely to feel that they received adequate support.

**Conclusions:**

It is important to explain the congenital nature of CL/P sufficiently and early. In addition, openness by the family about the diagnosis, rather than avoidance, may improve patients’ self-esteem. Sufficient support from family, health care providers, and significant others is needed for patients to develop adequate self-esteem.

## Background

According to nationwide monitoring by the Japan Association of Obstetricians and Gynaecologists, the annual incidence of congenital abnormalities in new-borns is 3–5% [1]. One of the most prevalent visible morphologic abnormalities is cleft lip and palate (CL/P). Compared with the CL/P incidence of 1 in 800 in Europe and the United States, the incidence in Japan is higher at 1 in 500 [2].

Treatment for CL/P proceeds in stages and begins 3–6 months after birth with the attending physician first discussing the situation with the parents. A therapeutic plan is introduced to the parents, and the parents and physicians come to an agreement. Team-based care is common in Japan and includes oral surgeons, plastic surgeons, otolaryngologists, orthodontists, speech therapists, and others, but parents and physicians remain at the centre of treatment decisions, not just during infancy, but continuing into adulthood.

Some healthcare providers previously believed that disclosing the diagnosis might cause greater harm than good to their patients, especially children. Consequently, professional appeals to “do no harm” resulted in nondisclosure or partial disclosure of health-related information [3]. Healthcare providers believed that they should protect paediatric patients from their diagnoses and prognoses, believing that young children could not comprehend concepts such as death, serious illness, or medical treatment. A paradigm shift in medicine toward greater patient autonomy and shared decision-making led to a more open disclosure policy with children in the 1970s and 1980s. Subsequent studies have indicated that young children could achieve a basic understanding of complex medical phenomena [3].

Clarifying the explanation of CL/P to patients and the effects of this condition is important. However, in Japan, virtually no study has investigated how withholding the truth or avoiding clear explanation is perceived and interpreted by people with CL/P. Walesky-Rainbow and Morris also raised these concerns in Europe and the United States, but it has not been examined further in these regions [4]. Studies of the psychosocial aspects of CL/P in Japan have focused primarily on the process of acceptance by the child’s mother [5], disclosure of the prenatal diagnosis of CL/P [5], and other factors concerning the parents, especially the mother. For the patients themselves, very few studies have been conducted on the parent–child relationship and personality traits [6].

In Europe and the United States, numerous studies have examined psychosocial factors in patients with CL/P and report that self-perception [7], self-esteem [8], and adjustment [9] are considered important indicators of psychosocial health in these patients. In particular, adolescents often face severe stigma, such as teasing or bullying, and low self-esteem and self-concept have been reported [10]. With respect to Japanese cultural differences, Hirose suggested that the Japanese characteristic of avoiding direct discussion of congenital facial defects has prevented the accumulation of research [11]. There are cultural factors in Japan that may underlie the unclear explanations and truth avoidance. Truth-telling means not hiding the truth and not telling a lie; it means explaining the entire treatment process rather than just naming the condition. Japanese culture includes a descriptive style that encourages ambiguity in speech and avoidance of clarity, and a culture of shame and ridicule related to facial appearance. Japan is a racially homogenous nation with a culture that respects harmony and places great value on sameness. This results in extreme sensitivity to differences, and differences in facial appearance readily lead to exclusion. People fear that they too will be excluded if they discuss CL/P openly. Because surgical treatment helps decrease the visible appearance of CL/P, people in Japan choose to believe the condition never existed. Finally, Japanese culture includes a concept called *sekentei*, which concerns public image and respectability. *Senkentei* favours avoidance of shameful behaviour, and promotes concern over public perception, fear of social criticism, self-restraint in deference to others, and privacy within the family [12].

In the present study, we used a questionnaire survey to clarify when and how people with CL/P became aware of their condition, their understanding of the condition, and the perceived effects of withholding the truth. Our objective was to examine the relationships between the timing of their awareness/disclosure of CL/P, their relationships with family and physicians, and self-esteem.

## Methods

This study was approved by the Ethics Committee of the Graduate School of Medicine and Faculty of Medicine, The University of Tokyo (Approval number 1277).

### Participants

There were three inclusion criteria for patients: (1) adult ≥ 20 years old; (2) no physical complications or comorbidities (exclusion criteria: intellectual delay or disability, physical disability, and congenital disease such as Pierre Robin syndrome); and (3) knowledge of the name and nature of the diagnosis (i.e., aware of the congenital nature). Criterion (3) was particularly carefully verified in participants. All participants gave their informed consent after receiving a written explanation of the purpose and content of the study. Knowledge of the CL/P diagnosis was verified based on answers to the following questions: Question 1: Your concern during the hospital examination was your lips, but what specifically did you have questions about? If the subject did not include, cleft lip and palate in their answer, they were not enrolled in the study. Question 2: What type of illness do you believe affects your mouth, or what do you believe the underlying cause is? Subjects were enrolled if they stated that they were born with the condition, the lip was split from birth, or the lip had never fused, indicating some understanding of the congenital nature of CL/P.

### Subject recruitment

Participants were recruited by three routes as follows:

**Table 1:** **Participant sociodemographic characteristics**

	Total	
	No. patients (n = 71)	(%)
Sex	20	(28.2)
Male	51	(71.8)
Female		
Age, mean ± SD (range, 20–75 years)	33.8 ± 7.5	
CL/P type		
Unilateral CL/P	33	(46.5)
Bilateral CL/P	19	(26.8)
Unknown CL/P	8	(15.4)
Cleft lip (unilateral)	5	(7.0)
Cleft lip (unknown)	1	(1.4)
Cleft palate	1	(1.4)
No answer	4	(5.6)
^a^Recruitment		
Hospital	10	(14.1)
Patient association	27	(38.0)
Snowball sampling	34	(47.9)
^b^Self-esteem score (range, 10–50)	33.0 ± 4.1	
Self-stigma score (range, 3–12)	6.29 ± 1.8	
Disclosure score (range, 3–12)	10.7 ± 6.2	

CL/P, Cleft lip and palate; SD, standard deviation; No., number.

^a^Study participants were recruited as follows: during routine examination (hospital route), through the Association of Cleft Lip and Cleft Palate in Japan (patient association route), or by acquaintance with an investigator or other participant (Snowball sampling).

^b^Data presented as the mean ± standard deviation.

A) Hospital route: Subjects learned about the study from physicians at the Osaka University Dental Hospital in the CL/P specialty service and were selected by an attending physician during outpatient examination. The physicians were given the following guidelines for subject participation: 1) exclusion of patients immediately pre- and postoperatively; 2) inclusion of subjects felt to be mentally stable; and 3) subjects who appeared to understand the meaning of cleft lip and palate based on the two questions described earlier, as evaluated by the chief physician.
B) Snowball sampling: Subjects were acquainted with the researchers or other subjects, such as during hospitalisation or outpatient visits, on the internet, or at off-line meetings. The sample included four subjects known by the author, two direct acquaintances of the researcher, and two friends of the researcher’s relatives.
C) Patient association: The Association of Cleft Lip and Cleft Palate in Japan, one of the largest support groups for parents of children with CL/P, has a “cleft lip friend’s meeting,” which is held for patients with CL/P and an accompanying adult. The director of the group agreed to cooperate, and a questionnaire was distributed to the membership (subject with an accompanying adult).

From August 2005 to November 2010, questionnaires were distributed to 11 participants by the hospital route, 36 participants by the snowball sampling route, and 52 participants through the patient association. Ten of 11 participants (90.9%) from the hospital route, 35 of 36 participants (97.2%) from snowball sampling, and 27 of 52 participants (51.9%) from patient meetings returned the questionnaires. One subject initially enrolled through the snowball sampling was 18 years old and thus did not meet the Japanese adult age cut-off criterion of ≥20 years; the patient was excluded. The patient attributes and characteristics are shown in Table 1.

### Survey and measurements

A Cronbach coefficient alpha was calculated for each survey for the 71 included participants.

1. Self-esteem was measured using Rosenberg’s scale [13] translated into Japanese by Yamamoto et al. [14]. This scale comprises 10 questions with five possible responses. Scoring differs from the original Rosenberg scale (10 items on a 4-point scale), but the Japanese translation is one of the most commonly used self-esteem scales in Japan [15, 16]. The higher the score, the higher the self-esteem (Cronbach coefficient alpha, 0.89).
2. Self-stigma was measured based on the self-stigma scale described by Link et al. [17]. Three questions with four possible responses reflecting the study goals were asked. Higher scores represented greater self-stigma (Cronbach coefficient alpha, 0.70).
3. Each subject’s experience of understanding the diagnosis at different time periods was evaluated as follows: elementary school, 6–11 years of age; junior high school, 12–15 years of age; and high school, 16–18 years of age. Elementary school was further divided into early (6–8 years of age) and late (9–11 years of age) elementary stages. Subjects were asked when and how they learned about CL/P and what explanations they received.
4. Each subject’s first awareness and experience of CL/P at different time periods was evaluated as follows: a) four questions on the concerns and understanding of the diagnosis; b) two questions on the physician-patient relationship; c) one question on family conversations about the diagnosis; d) six questions on stigmatising experiences, such as bullying, and the response to these experiences; and g) two questions on the parent–child relationship.
5. Based on a previous survey [18], the degree of disclosure to others about CL/P was determined using an original survey with three questions and four possible responses that considered the relationship between disclosure and self-stigma. The three questions were: “Can you talk about your condition being congenital?”; “Can you state specifically that it is a congenital anomaly?”; and “Even when not asked, can you talk about your condition when necessary?” The higher the score, the greater the subject’s tendency to disclose his or her CL/P condition to others (Cronbach coefficient alpha, 0.80).
6. Eight questions measured demographic characteristics as follows: age, sex, educational background, marital status, employment status, and socioeconomic group.

### Statistical analysis

#### Evaluation of patients’ understanding of the CL/P diagnosis by time period

The t-test was used for continuous data, and the chi-squared test was used for cross tabulation. Multiple comparisons (Kruskal-Wallis and Bonferroni correction) were performed for the 71 participants to evaluate their understanding of CL/P at different time periods (before and during elementary school, junior high school, and high school). The data were also subjected to regression analysis with self-esteem category as the dependant variable.

### Self-esteem categorisation

Participants were divided into high, middle, and low groups by self-esteem score and comparatively analysed, a technique employed in numerous fields [16]. Typically, when dividing participants into groups, a cut-off of the mean ± 1.5 standard deviation (SD) is sometimes used or participants are divided into three equal-sized groups [19]. Because the self-esteem scores in this study were higher than those in other Japanese studies of the same age groups [20], a cut-off point of the mean ± 1.5 SD and dividing the participants into three equal groups were considered inappropriate. After considering the mean and median values, the distributions reported in previous studies, and the interview results, we divided the participants into three groups based on the self-esteem scores as follows: high self-esteem group, total score ≥ 36 points (25 participants); middle self-esteem group, 29 < total score < 36 (26 participants); and; low self-esteem group, total score ≤ 29 points (20 participants). The group allocations were based on a previously published protocol [18].

## Results

The sociodemographic characteristics of the participants are shown in Table 1. The majority of participants (71.8%) were women, and the mean age of the group was 33.8 years. Unilateral CL/P was the most common diagnosis (46.5%), but some participants were unsure of whether their CL/P was complete or incomplete, or unilateral or bilateral.

### Age of CL/P disclosure and patient response

As shown in Table 2, 67 participants (94%) noticed that their nose and mouth were different from those in others during pre-elementary or elementary school. Upon realising the difference, 38 (53.5%) asked another person why. Most participants asked their parents, and 28 (39.4%) were told that the condition was congenital, but 17 (24.0%) were given untrue explanations, such as an injury resulting from a fall out of bed or outside, or injury due to the umbilical cord being wrapped around the head.

**Table 2:** **Participants knowledge of their cleft lip and palate (n = 71)**

	n	(%)
*Age when participants noticed their mouth/lip was different*		
Pre-elementary school (0–5 years)	30	(42.3)
Early elementary school (6–8 years)	29	(40.8)
Late elementary school (9–11 years)	8	(11.3)
Junior high school (12–15 years)	4	(5.6)
*Action upon noticing their CL/P*		
Asked someone	38	(53.5)
Did not ask anyone	28	(39.4)
Other response/No answer	5	(7.0)
*Explanation provided by others upon asking (multiple answers)*		
Condition name	11	(15.5)
Identified as a malformation	19	(26.8)
Congenital etiology	28	(39.4)
Cause	8	(11.3)
False explanation	17	(24.0)
*Age when participants learned the name, cause, and congenital name of malformation*		
Pre-elementary school	9	(12.7)
Early elementary school	10	(14.0)
Late elementary school	15	(21.1)
Junior high school	11	(15.5)
High school	4	(5.6)
Post-high school (≥19 years)	22	(30.1)
^*a*^ *Manner that participants obtained the above information (n = 70)*		
Told by someone	40	(57.1)
Learned on own	22	(31.4)
Learned by chance	8	(11.4)

CL/P, cleft lip and palate.

^a^Participants learned of their own CL/P from the following individuals: mother (n = 24), attending physician (n = 3), other physician (n = 2), father (n = 2), friend (n = 2), or other (n = 7).

### Frequency of discussion on CL/P with others and underlying motivations

Among the participants who noticed that they had CL/P, 28 (39.4%) did not ask anyone about their condition. In the free description column of the questionnaire, nine participants stated that they felt the topic was taboo based on their parents’ demeanour and on daily exchange with family members. One patient stated that “it was hopeless even to ask about that face,” (4) and “that my parents would feel sad if I asked about my diagnosis” (4).

### Manner of CL/P disclosure and associated self-esteem

Thirty-four (47.8%) participants knew that their condition was congenital by the end of elementary school (11 years of age), while 22 (30.1%) knew after age 19 years. Twenty-four participants learned about their CL/P from their mothers; three from the attending physician; and two from their father. However, several participants reported learning about their CL/P in an undesirable and traumatic way. For example, one participant became aware during the birth of her child; the obstetrician diagnosed her child with CL/P and informed the mother of her own CL/P simultaneously. A total of 22 (31%) participants learned about CL/P on their own using books and the Internet, and 19 participants learned after 19 years of age, indicating that many participants were unaware of their condition for a long period.

As shown in Table 3, the multiple comparisons (Kruskal–Wallis, Bonferroni correction) revealed that participants who learned about their CL/P while in elementary school (6–11 years of age; mean score, 3.2 ± 0.82) were more likely to believe that it was the best time than were participants who learned about it after high school. Two participants wrote that “Being told too early is not good,” and one replied that “The time did not matter,” but most participants did not consider themselves too young to understand CL/P during kindergarten or elementary school. The mean scores for participants who were first informed during junior and senior high school were significantly lower at 2.82 ± 0.41 (p < 0.001) and 1.92 ± 0.63, respectively.

**Table 3:** **Participants’ initial awareness of and response to CL/P according to time period**

Period when participant learned about CL/P	n	Satisfaction score ^a^	p-value ^b^
Before or during elementary school	34	3.24 ± 0.8	0.74*
Junior high school	11	2.82 ± 0.4	0.01**
High school/post-high school	26	1.92 ± 0.6	0.000***

CL/P, cleft lip and palate.

^a^Participants were asked to respond to the following statement: “I am satisfied that I came to know about my CL/P condition during this time period,” with one of four responses (strongly agree [4 points] to strongly disagree [1 point]). A higher score indicated greater satisfaction by the respondent.

^b^Significance defined as p < 0.05 and determined by the Kruskal–Wallis test as follows: *comparing elementary and junior high school periods, **comparing junior and high school/post-high school periods, and ***comparing elementary school and high school/post-high school periods.

Table 4 shows the participants’ experiences during each time period. Even during high school (16–18 years of age), approximately 38% did not understand that their facial scarring and articulation disorders were due to CL/P. Similarly, only 48% of participants who visited a hospital or were hospitalised during high school understood the reason and the nature of the treatment.

**Table 4:** **Participants’ answers to questions about having CL/P by time period (n = 71)** ^**a**^

	Early elementary school (6–8 years)		Late elementary school (9–11 years)		Junior high school (12–15 years)		High school (16–18 years)		≥19 years		Never	
	n	%	n	%	n	%	n	%	n	%	n	%
Awareness and understanding of CL/P												
I was worried about the scars around my mouth and the shape of my nose.	35	(50.0)	43	(61.4)	47	(67.1)	50	(71.4)	46	(65.7)	1	(1.4)
I was worried about my speech.	26	(36.6)	25	(35.2)	32	(45.1)	28	(39.4)	24	(33.8)	19	(26.8)
I understood that the scars around my mouth and my speech were due to a medical condition.	22	(31.9)	33	(47.8)	43	(62.3)	43	(62.3)	50	(72.5)	2	(2.9)
I understood the reason for the hospital visits and admissions.	13	(18.3)	20	(28.2)	35	(49.3)	37	(52.1)	42	(59.2)	7	(9.9)
*Relationship with physician*												
I actively asked my doctor questions.	0	(0.0)	2	(2.9)	2	(2.9)	11	(15.7)	23	(32.9)	23	(32.9)
I think my doctor knows my feelings and hopes well.	6	(8.5)	5	(7.0)	7	(9.9)	16	(22.5)	17	(23.9)	29	(40.8)
Family conversations about CL/P												
It was natural to talk about my medical condition with my family.	11	(16.7)	17	(25.8)	17	(25.8)	18	(27.3)	23	(34.8)	24	(36.4)
Stigmatizing experiences												
Someone pointed out my face or speech.	46	(64.8)	52	(73.2)	45	(63.4)	27	(38.0)	23	(32.4)	2	(2.8)
It was hard to have my face or speech pointed out.^b^	16	(34.8)	21	(40.4)	27	(60.0)	9	(33.3)	6	(26.1)	-	-
Someone made fun of my face or speech.	33	(47.1)	44	(62.9)	30	(42.9)	8	(11.4)	6	(8.6)	17	(24.3)
It was hard to be mocked about my face or speech.^b^	13	(39.4)	21	(47.7)	19	(63.3)	0	(0.0)	1	(16.7)	-	-
Someone used abusive words or violence.	25	(35.2)	35	(49.3)	33	(46.5)	10	(14.1)	6	(8.5)	24	(33.8)
It was hard to experience abusive words or violence.^b^	14	(56.0)	18	(51.4)	21	(63.6)	3	(30.0)	1	(16.7)	-	-

Participants were asked to circle all periods when the answer was yes. The number of participants circling each item was calculated for each period.

^a^The number (n) differs for each question due to the omission of missing values.

^b^Percentages were calculated based on the total population of people with stigmatizing experiences.

**Table 5:** **Significant differences in survey scores and responses between the high and low self-esteem groups**

	High SE group (n = 25)	Low SE group (n = 20)	
	Mean ± SD	Mean ± SD	p-value ^d^
Self-esteem score (range, 10–50)	41.0 ± 3.8	22.5 ± 4.8	0.000
Self-stigma score (range, 3–12)	5.0 ± 0.9	7.7 ± 2.8	0.006
Disclosure of CL/P to others (range, 3–12)	12.1 ± 1.4	7.0 ± 3.0	0.000
I was raised not to be conscious about my CL/P^a^	12 (48.0%)	16 (80.0%)	0.045
Period during which participants received sufficient support from someone important to them (range, 1–5)^b,c^	1.8 ± 0.8	1.0 ± 0.7	0.054
Period during which participant actively asked doctors questions about CL/P (range, 1–5)^b,c^	1.2 ± 1.0	0.6 ± 0.4	0.079

SE, self-esteem; SD, standard deviation; CL/P cleft lip and palate.

^a^Number (percentage) of participants who answered “yes” on this survey question.

^b^Responses from 45 participants (25 high SE and 20 low SE subjects) who consulted someone upon experiencing difficulty with their CL/P.

^c^For each period (early elementary school, 6–8 years; late elementary school, 9–11 years; junior high school, 12–15 years; high school, 16–18 years; and ≥19 years), 1 point was given if the subject answered that he/she was treated well, for a maximum of 5 points.

^d^Significance defined as p < 0.08.

### Family and physician relationships

As shown in Table 4, after reaching adulthood, only 32.9% of participants asked their doctors about CL/P, and less than 40% felt that they currently had good communication with their doctors. Only 17 patients (23.9%) felt that their doctors understood their feelings and goals after age 19 years, and 29 patients (40.8%) answered no to the same question.

In general, the participants enjoyed a good relationship with their parents as children, with 84.5% (60) reporting a good or somewhat good relationship, and trust with their parents. However, the patients also reported a tendency to avoid frank discussion of CL/P within the family or openly discuss problems and concerns related to the diagnosis. Overall, 37 (52.1%) of participants reported being raised not to be self-conscious of their CL/P.

As shown in Table 4, a high percentage of patients reported experiencing stigma due to teasing during late elementary school, which peaked during junior high school. Most participants discussed their anxieties with their mothers, but more than 29 participants (40%) experiencing stigma during early elementary school did not discuss the problem with anyone. Several participants who did seek outside counsel stated that the support was insufficient. As participants matured, more people were available to provide advice, but 60–80% of subjects still did not discuss the matter with anyone else. Among the reported reasons, participants expressed resignation that the parents would be able to intervene or would dismiss their concerns. Participants also wished to avoid saddening their parents over their experienced of being bullied.

### Comparison between self-esteem groups

No significant differences were observed between the self-esteem groups in participant characteristics, the parent–child relationship, the incidence of teasing, and social stigma. However, as shown in Table 5, the high self-esteem group showed significantly lower self-stigma and significantly greater disclosure of CL/P to others than the other groups. In addition, during periods of social difficulty, for example when being bullied in elementary school, individuals with high self-esteem were more likely to receive sufficient support from someone important to them. The regression analysis was also performed using self-esteem as the dependent variable; however, there were no statistically significant differences between the participant groups.

## Discussion

### Participant characteristics

Ours is one of the few studies assessing the disclosure to and understanding of people with the congenital condition CL/P. In Japan, the psychosocial study of CL/P is extremely sensitive. Even in a hospital setting, when adult subjects attended with a parent, several parents secretly refused to allow participation because they had not yet fully explained CL/P to their son or daughter (aged 20 and older). In Japan, it is not unusual for parents to accompany their adult children to medical appointments, even those older than 20 years of age. Parents often feel guilt over the congenital nature of CL/P and also wish to follow the long-term progress. The parents’ permission was required by the chief physician overseeing the study if the parent was present with their child, even if the child was older than 20 years of age and mentally able to consent. There were also several cases in which the attending physician could not determine whether the subject understood the nature of his or her condition.

Compared with the total population of patients with CL/P in Japan, 71 participants is certainly not large, which is a limitation of this study. However, considering that at least 20 parents declined to allow their children to participate in the study to protect them, collecting detailed data from even 71 Japanese participants is quite exceptional.

### Manner of disclosure and family conversations

It appears that participants did not receive sufficient explanation when they asked their parent(s) about their CL/P. Many participants did not fully understand their condition until entering adulthood. The reasons underlying this inadequate explanation from the parents may include anticipation and anxiety about potential discrimination that their child may experience, such as bullying and stigmatisation. We speculate that the expectation of parents that they can deny the existence of CL/P after surgical repair favours hiding the condition as a strategy of coping with social stigma [21]. The parents hide the details of the condition not only from surrounding people but also from their children, likely out of concern. They fear that their child may be hurt from knowing the truth, or they fear that their child may offend others due to the congenital diagnosis. Currently, the manner in which subjects are told about their diagnosis depends not on physicians but on parents, and many parents fail to disclose the truth for a long time [22]. This may reflect the lack of support on how to explain the condition to their children; therefore, children grow up without understanding CL/P.

Notably, the participants in this study desired a sufficient explanation at an earlier stage. In the United States, the idea that even a child has the basic human right to full knowledge of his or her medical condition is taken for granted. For example, in a study of children with leukaemia, investigators found that disclosing information such as the name of the disease and the treatment plan relieved anxiety in the children, increased their trust in healthcare providers, and enhanced the ability to self-control the disease [23]. Physicians play a role in truth-telling with patients with CL/P, but Saiki-Craighill found that many doctors were generally more conservative in this respect than parents, even though Japan adheres to the United Nations Declaration of the Rights of the Child, as does the United States [24]. The resistance of physicians to discussions about the diagnosis may be an important factor in patients’ awareness and acceptance of their diagnosis. For people with CL/P, who require prolonged treatment, understanding their current status and the necessity for treatment are important. One report in Japan observed that many people with CL/P discontinue corrective treatment, indicating that explanation of medical support to people with CL/P may be necessary to prevent drop-out [25]. According to a survey conducted in Japan on doctor–patient communication, patients with depression (n = 2020) reported that 55.0% of their interactions were satisfactory, whereas 14.3% were unsatisfactory [26]. Because CL/P and depression are different, a direct comparison between these studies is difficult. However, in cases of CL/P, it appears that the communication between patients and doctors is insufficient.

### Avoidance of CL/P discussion in the family and its impact and importance

In our study, approximately 52% of participants reported being raised not to be self-conscious of their CL/P. However, regardless of whether their avoidance behaviour stems from care or affection for the child, when parents insist on pretending that the CL/P does not exist, talking about the diagnosis may become taboo in the family. When the child recognises a potential difference in the facial appearance compared with others, he or she may be unable to discuss the matter with family and may not seek support. The majority of participants experienced the greatest amount of teasing related to their CL/P during junior high school, but many did not consult anyone. One reason reported by the participants was the resignation that seeking help would be ineffective and would worry their parents. Scambler & Hopkins reported that when suffering is hidden, stigma is internalised, and self-stigma may result [27]. Similarly, Matsumoto et al. stated that when suffering is hidden, individuals have no psychological strength to face their CL/P [28]. Approximately 80% of participants in the low self-esteem group reported being raised not to be self-conscious of their CL/P compared with 48% in the high self-esteem group; yet, self-stigma was significantly higher in the low self-esteem group than in the high self-esteem group. These results support those reported by both Scambler et al. and Matsumoto et al. [27, 28].

### Clinical implications

Our results suggest that people with CL/P routinely have their facial difference pointed out repeatedly over a long period beginning at infancy. They experience stigma and feel uncertainty over lacking full knowledge about their diagnosis. This situation may seem trivial, but it slowly degrades affected individuals, deprives the child of self-esteem and self-affirmation, and can lead to appreciable psychological trauma. Ensuring safety and security is vital in the initial treatment of psychological trauma [29]; therefore, it is important that people with CL/P receive warm support from their family and significant others, and that they find a safe place to relax. Miyaji stated that those who have no choice but to recognise their difference find safety and security with their peers [29]. Therefore, interaction with other patients with CL/P is considered very important, and the patient’s medical team should provide this emotional support. Additionally, the medical team plays a crucial role in explaining the diagnosis to subjects and promoting their understanding of CL/P, as well as providing psychological support and intervention. Omiya et al. reported that explanations must be tailored to the patient’s level of understanding, especially concerning age [18]. Multiple explanations throughout a patient’s lifetime are necessary, and the patient-care team must choose the best person(s) to initiate and continue those discussions. More research is needed on the training of both medical personnel and parents to better communicate the nature of CL/P to patients and to overcome the cultural barriers currently existing in Japan.

### Study limitations and challenges

This study is biased in its study population, which was carefully selected by attending physicians who assessed the subjects’ psychological status and treatment history. The study participants had higher-than-average self-esteem scores compared with the scores in an earlier study [30]. This suggests that the participants in our study were in a better psychological status, and therefore, subjects who were suffering from more serious psychosocial problems may have been underrepresented. In Japan, the incidence of CL/P is slightly higher in men than in women, but 71.8% of our subjects were female and 28.2% were male. The difference between our ratio and the prevalence could influence the results; therefore, caution is warranted when generalising the implications.

Because the subjects were surveyed retrospectively, with some experiences dating back several decades, ideally, parents and healthcare providers should also have been surveyed to improve the credibility of the study, understand the complete clinical picture, and obtain a multifaceted view. With the medical advances since the time of surgery in the subjects, it may currently be easier for parents to explain the condition.

Finally, our study was specific to the Japanese CL/P population and may not be applicable in other countries.

## Conclusion

Our participants reported long-standing difficulty with understanding their condition. Their stigmatising experience rates were high, particularly during junior high school age. Although the stigmatising experiences were extremely painful, many patients hid their suffering, making it more difficult to obtain social support. The participants felt that their families avoided the topic of their diagnosis. Within the family, talking about the condition, rather than considering it taboo, would likely make it easier for patients with CL/P to discuss their troubles and worries. Participants with high self-esteem felt that they received adequate support and were open with others regarding their congenital condition. It is important to explain the congenital nature of CL/P sufficiently and early. Sufficient support from family, health care providers, and significant others is necessary for patients to develop adequate self-esteem.

## References

[1] Hirahara F (2007). Senten-ijyou monitaringu: Wagakuni to sekai no torikumi [Birth Defects Monitoring: Efforts of Japan and the World] (in Japanese). Acta Obstetrica et Gynaecologica Japonica.

[2] Natsume N, Kawai T, Kohama G, Teshima T, Kochi S, Ohashi Y, Enomoto S, Ishii M, Shigematsu T, Nakano Y, Matsuya T, Kogo M, Yoshimura Y, Ohishi M, Nakamura N, Katsuki T, Goto M, Shimizu M, Yanagisawa S, Mimura T, Sunakawa H (2000). Incidence of cleft lip or palate in 303 738 Japanese babies born between 1994 and 1995. Br J Oral Maxillofac Surg.

[3] Tse CY, Chong A, Fok SY (2003). Breaking bad news: a Chinese perspective. Palliat Med.

[4] Waleskyrainbow PA, Morris HL (1978). Assessment of informative-counselling procedures for cleft-palate children. Cleft Palate J.

[5] Nakanii M, Shinohara H, Moriguchi T (2005). Research on the actual state of disclosure in the prenatal notification of cleft lip and palate and recommendations for a desirable support system (in Japanese). Kawasaki Medical Welfare Journal.

[6] Kitamura Y, Ueda R, Tamaki A, Kawada S, Fujita H (2005). Self-esteem and its related variables in children with cleft lips and/or cleft palates. Bulletin of Research Institute, National Rehabilitation Center for Persons with Disabilities.

[7] Millard T, Richman LC (2001). Different cleft conditions, facial appearance, and speech: relationship to psychological variables. Cleft Palate Craniofac J.

[8] Gussy M, Kilpatrick N (2006). The self-concept of adolescents with cleft lip and palate: a pilot study using a multidimensional/hierarchical measurement instrument. Int J Pediatr Dent.

[9] Berger ZE, Dalton LJ (2011). Coping with a cleft II: factors associated with psychosocial adjustment of adolescents with a cleft lip and palate and their parents. Cleft Palate Craniofac J.

[10] Strauss RP, Ramsey BL, Edwards TC, Topolski TD, Kapp-Simon KA, Thomas CR, Fenson C, Patrick DL (2007). Stigma experiences in youth with facial differences: a multi-site study of adolescents and their mothers. Orthod Craniofac Res.

[11] Hirose T (1999). A literature review of psychosocial problems of children with cleft lip and cleft palate. Journal of Japanese Cleft Palate Association.

[12] Inoue T (2007). Sekentei no Kozo [the structure of sekentei] (in Japanese).

[13] Rosenberg M (1965). Society and the Adolescent Self-Image.

[14] Yamamoto M, Matsui Y, Yamanari Y (1982). The structure of perceived aspects of self. Jpn J Educ Psychol.

[15] Kaketa K, Nozaki T (2008). Evaluation of structured group encounter in training for teachers with ten years of experience at an academic seminar. Journal of Hokkaido University of Education.

[16] Matsuura N (2008). Empirical research on relationship between inattention, hyperactivity, Impulsivity, and self-esteem in inmates of a correctional facility (in Japanese). Human Developmental Research.

[17] Link BG, Struening E, Cullen FT, Shrout PE, Dohrenwend BP (1989). A modified labelling theory approach to mental-disorders - an empirical-assessment. Am Sociol Rev.

[18] Omiya T, Ito M, Yamazaki Y (2012). The process leading to affirmation of life with cleft lip and cleft palate: the importance of acquiring coherence. Jpn J Nurs Sci.

[19] Squires J, Bricker D, Potter L (1997). Revision of a parent-completed development screening tool: ages and stages questionnaires. J Pediatr Psychol.

[20] Menjyu N (2009). Analysis of the medical sociological several problems a mother of a granny birth has (in Japanese). Bulletin of International Buddhist University.

[21] Conrad MM, Pacquiao DF (2005). Manifestation, attribution, and coping with depression among Asian Indians from the perspectives of health care practitioners. J Transcult Nurs.

[22] Sado A, Ishii M, Ishii Y, Moriyama T, Morita K, Gunji A, Imaizumi F, Murase K, Takahashi Y, Enomoto S (2001). Clinical study on telling patients with cleft lip and cleft palate the name of their disease (in Japanese). Journal of Japanese Cleft Palate Association.

[23] Leikin SL (1981). An ethical issue in pediatric cancer care: nondisclosure of a fatal prognosis. Pediatr Ann.

[24] Saiki-Craighill S (1998). The attitude of pediatric oncologists regarding disclosing the nature of the illness to children. J Child Health.

[25] Ito K, Matsuura S, Ohta K (1993). A survey on cleft lip and cleft palate patients who discontinued corrective treatment at Orthodontic Dentistry of Hiroshima University Dental Hospital. Journal of Japanese Cleft Palate Association.

[26] Sawada N, Uchida H, Watanabe K, Kikuchi T, Suzuki T, Kashima H, Mimura M (2012). How successful are physicians in eliciting the truth from their patients? A large-scale internet survey from patients’ perspectives. J Clin Psychiat.

[27] Scambler G, Hopkins A (1986). Being epileptic–coming to terms with stigma. Sociol Health Illn.

[28] Matsumoto M (1999). Youbou-no Jko Jyuyou-Koshin/Kogairetu no Baai (Self-acceptance of looks-in case of cleft lips and cleft palate). Gendai Bunmei Kenkyu.

[29] Miyaji N (2005). Torauma no Iryojinruigaku (Medical anthropology of trauma) (in Japanese).

[30] Toyota K, Matsumoto T (2004). Factors relating to self-esteem in university students. (Daigakusei-no Jisonshin to Kanren-suru Shoyouin ni Kansuru Kenkyu). The Bulletin of the Institute of Human Sciences, Toyo University.

